# Striking a balance: does nitrate uptake and metabolism regulate both NO generation and scavenging?

**DOI:** 10.3389/fpls.2013.00288

**Published:** 2013-07-30

**Authors:** Luis A. J. Mur, Kim H. Hebelstrup, Kapuganti J. Gupta

**Affiliations:** ^1^Institute of Biological, Environmental and Rural Science, Aberystwyth UniversityAberystwyth, UK; ^2^Department of Molecular Biology and Genetics, Aarhus UniversitySlagelse, Denmark; ^3^Department of Plant Sciences, University of OxfordOxford, UK

Nitric oxide (NO) influences many aspects of plant development and responses to stress.The concentration of NO can play an important role in influencing its action (for example, in stomatal regulation; Wilson et al., 2009) so that the mechanisms through which NO content is modulated must be an important facet of NO research. Whilst NO generation mechanisms are clearly important, NO removal is of equal relevance, especially as plants will be continually exposed to NOx (NO + NO_2_) gases derived from soil microbial activity (Mur et al., 2013). Establishing and regulating a poise between NO generation, NO fumigation from external sources and NO scavenging, which also needs to be flexible enough to change in response to a variety of physiological cues, is an under-considered aspect of plant NO biology.

## How are no generation mechanisms integrated and regulated?

Initially, many sought to find an equivalent to the mammalian Nitric Oxide Synthase (NOS) enzymes in plants. NOS is a cytochrome P450-like enzyme which oxidizes arginine to citrulline to generate NO (Gorren and Mayer, 2007). However, the existence of this enzyme in higher plants is still debatable (Frohlich and Durner, 2011) and is mainly based on pharmacological evidence and assays for NOS-like enzyme activity reviewed by Frohlich and Durner (2011) and Mur et al. (2013). In this context, it is also relevant that arginase mutants in Arabidopsis also displayed increased NO levels (Flores et al., 2008). However, NOS-activity has not been linked to a given gene. Resolution of this conundrum may derive from the observation that polyamine leads to NO production from Arabidopsis roots (Tun et al., 2006). As L-arginine is a precursor to polyamine biosynthesis, any perturbance of L-arginine metabolism would affect any polyamine-mediated NO generation mechanism and would explain the effects of NOS-inhibitors without needing NOS. Such a mechanism would be easily linked to the most well-characterized plant NO mechanism which is based on nitrate reductase (NR). NR acts by reducing nitrite to NO with NAD(P)H acting as an electron donor. NR-generated NO has been shown to regulate floral development, root formation, stomatal opening, and responses to biotic and abiotic stresses [reviewed in Mur et al. (2013)]. NR has high affinity for nitrate but switches to its lower affinity substrate nitrite to produce NO (Planchet et al., 2005). Therefore, NR requires high nitrite concentrations to produce NO; and a low pH is also required. Considering both NR and NOS-like NO generation mechanisms together it is possible to suggest some regulatory nodes. Thus, NO generation can be regulated at the level of NO^−^_3_ uptake via nitrate channels, post-translational modification of NR activity (Mur et al., 2013), influencing NO_2_ availability, pH and the expression and/or activity of any of the amino acid and polyamine biosynthetic enzymes. These potential regulatory mechanisms need to be systematically assessed.

Interestingly, NO^−^_3_ also plays a central role in anoxic/hypoxic NO generation. Under hypoxia, the resulting energy crisis leads to a decrease in pH which inhibits plasidal NiR, leading to NO^−^_3_ accumulation and NO production (Ferrari and Varner, 1971). NADH-dependent NO^−^_3_ reduction occurs at the mitochondrial inner membrane, via cytochrome c oxidase and/or reductase and possibly by alternative oxidase (AOX) leading to the production of NO and ATP (Stoimenova et al., 2007). NO production via this mechanism occurs below 1% oxygen with a Ki value of 0.05% (0.6 μ M) (Gupta and Igamberdiev, 2011). Again NO^−^_3_ and now also NADH are limiting factors and represent possible important regulatory steps and could be the mechanism through which nitrite is transported to mitochondria which is currently not known.

Regulating the availability of NO^−^_3_ also seems to be important in other less well-characterized NO generation mechanisms. NO may be generated in the peroxisome by a xanthine oxidoreductase (XOR) which can reduce NO^−^_3_ to NO (Del Rio et al., 2004). NO is also generated by a plasma membrane nitrite:NO reductase (NiNOR) where NO^−^_3_ is supplied by an apoplasmic, plasma membrane-bound NR.

## Balancing the equation: mechanisms of no removal

*In planta* NO content must represent the net of rates of production minus scavenging. These scavenging mechanisms must be highly efficient in order to maintain appropriate NO poise in crop species where the extensive use of nitrogen-fertilizers can result in external fumigation at rates that may be in excess of 20 nmol m^−2^ h^−1^ (Voldner et al., 1986; Benkovitz et al., 1996). Various means to reduce NO content have recently emerged; perhaps the most important being nonsymbiotic forms of hemoglobin (Hb). Oxygenated ferrous (Fe^2+^) Hb converts NO to NO^−^_3_ and becomes MetHb (ferric, Fe^3+^) (metamoglobin) form which is then reduced to oxygenated ferrous (Fe^2+^) by metamoglobin reductase (MetHb) (Hill, 2012). NO oxidation by Hb plays an important role in NO accumulation during stress (Hebelstrup et al., 2012; Mur et al., 2012) thus the regulation of Hb expression is vitally important to understanding how NO poise is established (Mur et al., 2013). It is highly relevant that NO^−^_3_ induces Hb (Wang et al., 2000) again showing how NO^−^_3_ regulates NO content, on this occasion by influencing NO scavenging.

Other enzymes through which NO effects are modulated include S-Nitrosoglutathione Reductase (GSNOR). NO reacts with glutathione GSH and forms S-nitrosoglutathione (GSNO), which represents a significant reservoir for NO (Sakamoto et al., 2002). GSNO levels are controlled by GSNOR with converts GSNO into glutathione and sulphinamide using NADH as electron donor. Thus, GSNOR represents a means through which NO signaling may be suppressed as has been demonstrated using GSNOR mutants (Feechan et al., 2005). Additionally, under aerobic conditions mitochondria are highly efficient NO scavengers (87% of supplied NO −180 pmol) (Gupta et al., 2005). Mechanistically, this has been linked to AOX via leaking electron flow from the electron transport chain to terminal electron acceptor oxygen or nitrite in the cytochrome pathway (Cvetkovska and Vanlerberghe, 2012).

**Figure 1:**
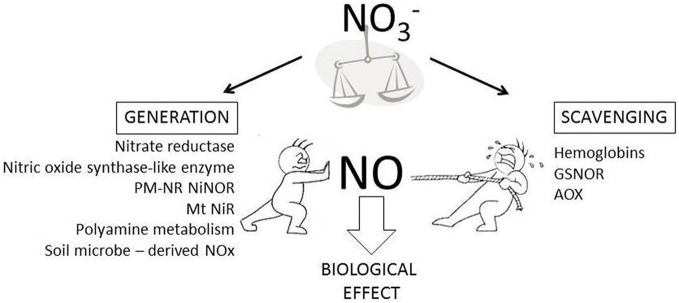
**In planta sources of NO generation and scavenging**. *In planta* NO content reflects the net effect of NO generation (shown as “push” in the Figure) and scavenging (shown as “pull” in the Figure) mechanisms. NO generation can involve the listed pathway (PM-NR NiNOR, plasma membrane associated nitrate reductase coupled to nitrite reductase; Mt NiR, mitochondrial nitrite reductase). The likely role of NO^−^_3_ in regulating *in planta* NO content is highlighted.

This opinion piece seeks to highlight some key questions regarding how *in planta* NO content is regulated (Figure 1). In developing these questions we have highlighted the role of NO^−^_3_. We suggest that understanding the regulation of NO^−^_3_ uptake, assimilation and processing into a myriad of biosynthetic pathways will be central to understanding how *in planta* NO content is established.
